# Benign appendiceal intussusception masquerading as caecal carcinoma intraoperatively in an adolescent male: a case report

**DOI:** 10.1097/RC9.0000000000001169

**Published:** 2026-10-21

**Authors:** Muhammad Wajid Siddique, Muhammad Zeshan, Ali Husnain, Muhammad Hanzla Shahzad, Muhammad Hassaan Javaid, Raghabendra Kumar Mahato

**Affiliations:** a Department of General Surgery, Ameer-ud-Din Medical College, Lahore, Pakistan; b Department of General Surgery, Lahore General Hospital, Lahore, Pakistan; c Department of General Surgery, Shifa Tameer-e-Millat University, Islamabad Pakistan; d Department of General Surgery, Gandaki Medical College Teaching Hospital and Research Center, Pokhara, Nepal

**Keywords:** appendiceal intussusception, case report, cecal carcinoma, ileocecal resection, intestinal intussusception

## Abstract

**Introduction:**

Appendiceal intussusception is a rare phenomenon that can mimic acute appendicitis, ileocecal intussusception, and even cecal malignancy, both clinically and intraoperatively. It becomes more difficult to recognize when the appendix is not seen on exploration.

**Case presentation:**

A 17-year-old man presented with 4 days of right iliac fossa pain, nausea and vomiting, anorexia, and 3 weeks of episodic right-sided abdominal pain. The patient had local peritonism, an Alvarado score of 8/10, leukocytosis, and nondiagnostic ultrasonography. We did not perform a contrast-enhanced computed tomography (CT) scan, in accordance with our institution’s practice. During laparotomy, the appendix was not found, even after cecal mobilization and a systematic search along the taeniae coli. A dimple on a thickened, edematous, and ischemic cecum raised suspicion of ileocecal intussusception or a cecal tumor. Limited ileocecal resection with primary ileocolic anastomosis was performed with informed consent from the family. Pathology showed complete appendiceal intussusception with transmural ischemic necrosis and acute inflammation without any evidence of malignancy, polyp, mucocele, or endometriosis. There was no evidence of malignancy in four regional lymph nodes.

**Discussion:**

A cecal dimple in the setting of an otherwise absent appendix should alert us to the diagnosis of appendiceal intussusception. A CT scan would be helpful for identifying the problem preoperatively if ultrasonography is inconclusive.

**Conclusion:**

Complete appendiceal intussusception can masquerade as cecal malignancy. Awareness of this rare entity may prevent unnecessarily extensive resection while ensuring appropriate treatment of ischemic bowel.

## Introduction

Intussusception of the appendix is a rare entity causing an appendiceal or cecal mass. Cases have been reported in 0.01% of appendicectomy specimens^[^1–5^]^. Organic causes include endometriosis, mucocele, adenoma, carcinoid tumor, and adenocarcinoma, although idiopathic cases have been encountered^[^6–8^]^. Abnormal motility, a long appendix, a wide appendiceal lumen, and/or a lax mesoappendix are other causes without an organic lesion. Clinically, the entity is similar to appendicitis; however, ultrasound shows a target or concentric ring appearance, and contrast-enhanced computed tomography (CT) shows an invaginated tubular structure at the cecal pole^[^2,3^]^. The entity may be identified intraoperatively, but this is not the only mode of identification. Here, we present an idiopathic intussusception of the appendix mimicking cecal carcinoma intraoperatively. This work has been reported in line with the SCARE criteria.

HIGHLIGHTS

- Complete appendiceal intussusception is a rare diagnostic entity that can mimic a cecal tumor.
- An absent appendix and a cecal dimple should raise suspicion for appendiceal intussusception during exploration.
- Nondiagnostic ultrasonography may warrant cross-sectional imaging when symptoms are atypical or prolonged.
- Histopathology confirmed idiopathic appendiceal intussusception with transmural ischemic necrosis and no structural lead point.
- Limited ileocecal resection provided definitive treatment and diagnosis while avoiding an oncologically unnecessary right hemicolectomy.
- Recognition of this condition may help tailor the extent of surgery to bowel viability and suspicion of malignancy.

## Case presentation

A 17-year-old man patient presented with a 4-day history of sudden-onset pain in the right iliac fossa, which was continuous and exacerbated by movement but partially relieved by self-medication with oral paracetamol and ibuprofen. This episode of acute pain was associated with an approximately 3-week history of intermittent pain in the right abdomen. The patient had been experiencing dysuria, diarrhea, and fever for 3 weeks, for which he took ciprofloxacin for 5 days at the primary health center. Pain score, urine culture, frequency or urgency of urination, highest temperature, stool frequency, and vomiting frequency were unknown. However, he experienced vomiting and loss of appetite in the 2 days before admission. His temperature on admission was 36.6^o^C. Thus, the prior fever was by history only.

He appeared to be ill. His pulse rate was 102/min, blood pressure was 90/70 mmHg, and respiratory rate was 26/min, with localized guarding, rebound tenderness, and a positive Rovsing’s sign in the right iliac fossa. The oxygen saturation level was not known. He became stable after fluid therapy, antibiotics, and analgesic administration. The vital signs post-resuscitation were not known.

### Investigations

White blood cell count was 15.0 × 10^3^/µL, and hemoglobin was 12 g/dL. Urinalysis revealed 3–5 pus cells per HPF without hematuria or proteinuria. Ultrasound revealed tenderness in the right iliac fossa, but there was no identification of an abscess, an appendiceal mass, a collection, a target sign, or definite visualization of the inflamed appendix. Based on an Alvarado score of 8/10, the patient had acute appendicitis with possible complications; no abscess was noted. Contrast-enhanced CT of the abdomen and pelvis was not performed, as it is not routinely obtained for teens with a high clinical suspicion of appendicitis at our facility. In hindsight, the long-standing symptoms, urinary complaints, diarrhea, and the nondiagnostic ultrasound made CT a suitable modality, as it would have helped characterize the cecal lesion and the intussusception of the appendix. CT enterography was not indicated.

### Management

The option for exploratory surgery was taken because of borderline hemodynamics, localized peritonitis, and the availability of resources within the institution. Upon extension to a Rutherford-Morison incision, the cecum was brought out. However, the appendix could not be located after checking the retrocecal, pelvic, and subcecal sites, as well as tracing the three taeniae coli to where they meet at the cecal pole. There was an area on the cecum that was dimpled, thickened, edematous, and ischemic, but had no perforation or contamination. The differential included ileocecal intussusception with a lead point and cecal malignancy; appendiceal intussusception was not recognized (Figs 1 and 2).

**Figure 1.**
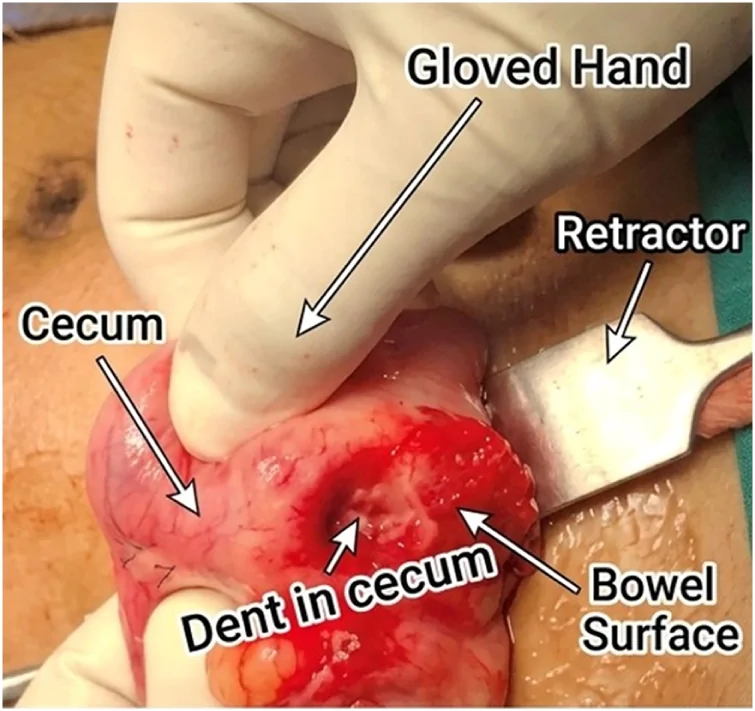
Inspection of the cecum revealed a shallow dimple at the appendiceal site.

**Figure 2.**
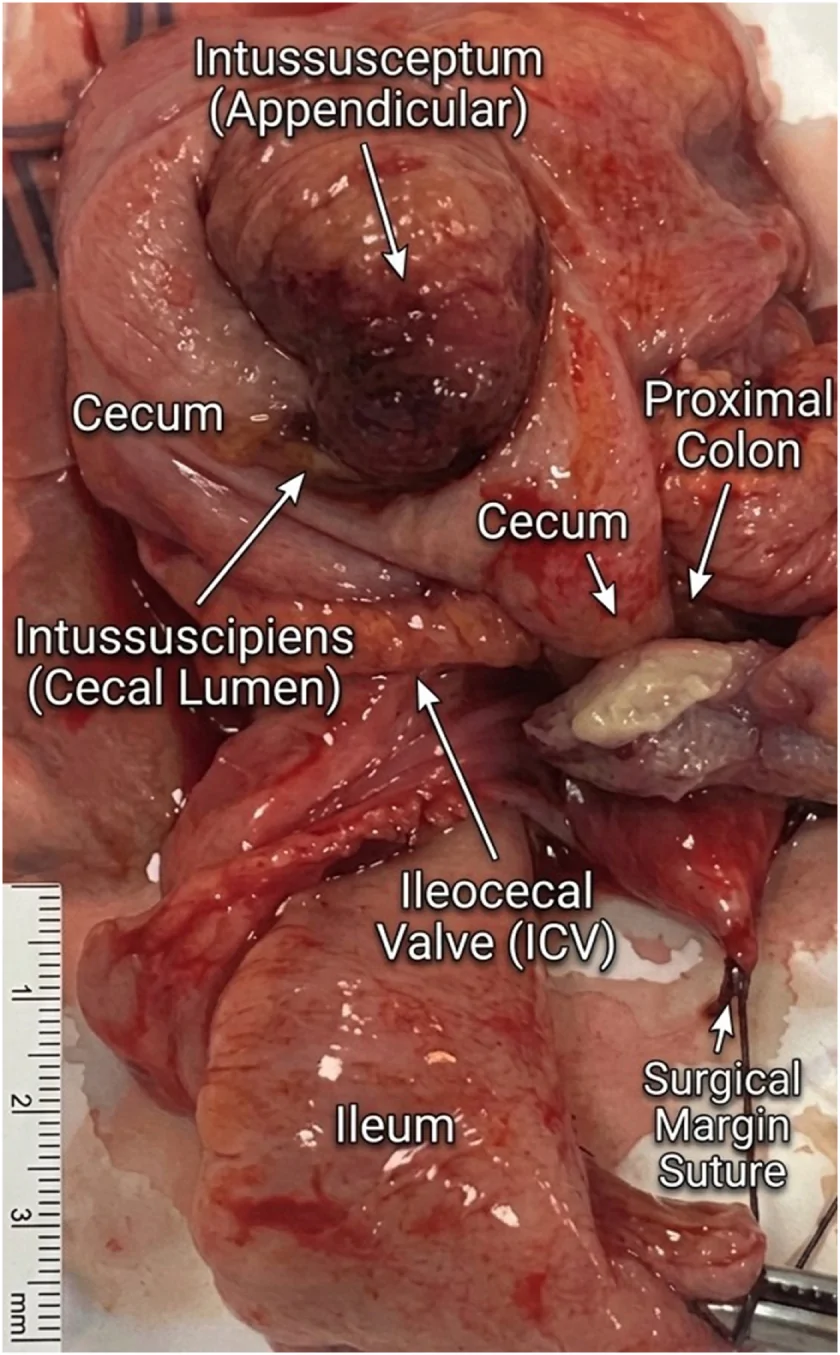
The appendix is completely invaginated into the cecal lumen, consistent with appendicular intussusception.

A combination of reduction and appendectomy could not be achieved because the cecal lesion had a high risk of ischemia and appeared as a mass, and an underlying neoplasm could not be completely ruled out. Manipulation and frozen sections were contraindicated, and the purpose was resection and diagnosis rather than oncologic resection. The family was informed about the unexpected findings and the possibility of malignancy, and the extended surgery was discussed. Ileocecal resection with end-to-end ileocolic anastomosis was performed via an extended Rutherford-Morrison incision. Right hemicolectomy with central vascular resection was not performed. The findings seemed localized, and resection was therefore appropriate, but no mention was made of vascular pedicle details. Macroscopically clear bowel margins were obtained, but were not considered oncologically equivalent to a formal hemicolectomy.

### Outcomes and follow-up

Macroscopic findings revealed complete invagination of the appendix within the lumen of the cecum. Pathological findings revealed severe ischemia, inflammation, ulceration, congestion, and hemorrhage without neoplasia, polyp, mucocele, or endometriosis. Reactive pericolic lymph nodes (four in total) were negative for malignancy (0/4), and proximal ileal and distal colonic margins were viable and pathologically normal. Idiopathic means the absence of well-known lead points.

His recovery was uneventful. He passed flatus and advanced to a soft diet on postoperative day three. No drain was inserted. No anastomotic leak or wound infection was observed clinically; therefore, imaging was not necessary. The patient was discharged on postoperative day seven. Follow-up visits were conducted at 2 weeks, 6 weeks, and 3 months postoperatively, with normal bowel movements and no recurrence.

## Discussion

Appendiceal intussusception is an exceptionally rare condition in which the appendix invaginates into the cecum, with an estimated incidence of approximately 0.01% during appendectomies^[^1,3,6^]^. The clinical features may appear similar to those of acute appendicitis or other types of intestinal intussusception; therefore, preoperative diagnosis can be challenging^[^1,3^]^. Some of the suggested risk factors include abnormal appendiceal motility, a long or hypermobile appendix, a wide appendiceal lumen, and laxity of the mesoappendix. The structural causes of intussusception include fecalith, mucocele, endometriosis, adenoma, and neoplasia. In our patient, histopathology revealed no evidence of a structural lead point, implying an idiopathic cause.

The previously published case reports demonstrate variation in presentation and management. For example, Petracic *et al*. described the case of a 14 years old boy with a cecal tumor-like mass identified intraoperatively; histopathology confirmed complete invagination of an inflamed appendix with fecalith formation^[^1^]^. Nan *et al*. described appendiceal intussusception after prior stump inversion appendicectomy, demonstrating one possible iatrogenic mechanism^[^4^]^. Tsukamoto *et al*. mentioned complete appendiceal intussusception in the absence of any organic lead point^[^3^]^. Shihab *et al*. described McSwain type V appendiceal intussusception with acute appendicitis in a 4-year-old patient^[^9^]^. Adane et al. reported on a 7-year-old male with ileocolic intussusception caused by appendiceal intussusception and acute appendicitis^[^7^]^. Features of our patient include age above infancy, lack of a lead point, significant ischemia, and an intraoperative appearance similar to that of a neoplasm. Comparison with the literature is given in Table 1

**Table 1:** Comparisons with other cases.

Case	Age/sex	Lead point/associated pathology	Imaging	Treatment	Outcome
Petracic *et al*^[^1^]^	14 y, M	Fecalith; complete appendiceal intussusception	US suggested a cecal mass	Surgical resection with primary anastomosis	Uneventful recovery; discharged
Tsukamoto *et al*^[^3^]^	Adult	None identified; idiopathic complete intussusception	Preoperative diagnosis reported	Appendectomy/resection according to operative findings	Uneventful recovery
Nan *et al*^[^4^]^	Adult, age NR	Iatrogenic; previous stump-inversion appendicectomy	Preoperative CT + colonoscopy	Elective resection	NR
Shihab *et al*^[^9^]^	4 y, F	Acute appendicitis; McSwain type V	NR	Operative management	NR
Adane *et al*^[^7^]^	7 y, M	Acute appendicitis with ileocolic and appendiceal intussusception	Imaging demonstrated intussusception	Surgical management	Uneventful recovery
Present case	17 y, M	None identified; idiopathic; severe ischemic necrosis	US nonspecific; CT not performed	Limited ileocecal resection with primary ileocolic anastomosis	Discharged POD 7; uneventful recovery

NR, not reported in the published abstract available to the authors.

Preoperative diagnosis is difficult. Ultrasound might show the classic “target” or “concentric ring” pattern, but these are not always evident^[^1,3^]^. The role of contrast-enhanced CT includes demonstrating invagination of the appendix or a tube-like structure at the cecal pole, which helps in the diagnosis^[^5^]^. A prolonged and atypical clinical presentation and a negative ultrasound in our patient might justify obtaining cross-sectional imaging, which would enable preoperative diagnosis^[^5^]^.

The absence of an appendix is a very helpful feature. Systematic inspection of the cecum and a search along the taenia coli can reveal an inverted appendix^[^1,3,6^]^. Hence, the presence of a dimple or mass on the cecum is an indication of appendiceal intussusception. Severe edema, ischemia, and a mass in our case suggested ileocecal intussusception or malignancy.

Management depends on the extent of intussusception, the viability of the bowel, the nature of the lead point, and suspicion of malignancy^[^1,3,6^]^. Reduction and appendectomy can be performed if the bowel is viable and there is no suspicion of malignancy, whereas resection may be indicated for ischemia or an uncertain lead point^[^1,6^]^. In this instance, an ileocecal limited resection was performed not only for treatment but also for diagnosis, and it was not oncologically equivalent to a right hemicolectomy. Pathology confirmed a benign idiopathic appendiceal intussusception.

Limitations include the lack of a preoperative CT scan, frozen section analysis, inadequate history, and a single-patient study. However, the absence of the appendix with a cecal dimple or mass should make us suspect appendiceal intussusception.

## Conclusion

The diagnosis of idiopathic appendiceal intussusception should be considered in the presence of a non-visible appendix and a cecal dimple or mass in a patient suffering from right iliac fossa pain. Viability assessment and exclusion of malignancy should be performed wherever possible. A conservative resection of the ileocaecal segment could be undertaken where there is clear intra-operative localization and convincing evidence of benignity, while oncological resection is the gold standard for cases of proven malignancy or where malignancy cannot be excluded. Good pre-operative imaging and proper communication with the family in unusual circumstances result in good outcomes.

## Data Availability

Data available on request from the authors

## Peer and provenance statement

Not commissioned; externally peer-reviewed
